# Antenatal Hypoxia Accelerates the Onset of Alzheimer’s Disease Pathology in 5xFAD Mouse Model

**DOI:** 10.3389/fnagi.2020.00251

**Published:** 2020-08-21

**Authors:** Guofang Shen, Shirley Hu, Zhen Zhao, Lubo Zhang, Qingyi Ma

**Affiliations:** ^1^Department of Basic Sciences, The Lawrence D. Longo MD Center for Perinatal Biology, Loma Linda University School of Medicine, Loma Linda, CA, United States; ^2^Department of Physiology and Neuroscience, Center for Neurodegeneration and Regeneration, Zilkha Neurogenetic Institute, Keck School of Medicine, University of Southern California, Los Angeles, CA, United States

**Keywords:** Alzheimer’s disease, 5xFAD mouse, antenatal hypoxia, cognitive impairment, synapse loss, gliosis

## Abstract

Alzheimer’s disease (AD) is a chronic neurodegenerative disorder associated with cognitive impairment and later dementia among the elderly. Mounting evidence shows that adverse maternal environments during the fetal development increase the risk of diseases later in life including neurological disorders, and suggests an early origin in the development of AD-related dementia (ADRD) *in utero*. In the present study, we investigated the impact of antenatal hypoxia and fetal stress on the initiation of AD-related pathology in offspring of 5xFAD mice. We showed that fetal hypoxia significantly reduced brain and body weight in the fetal and the early postnatal period, which recovered in young adult mice. Using spontaneous Y-maze, novel object recognition (NOR), and open field (OF) tasks, we found that antenatal hypoxia exacerbated cognitive decline in offspring of 5xFAD compared with normoxia control. Of interest, fetal hypoxia did not alter intraneuronal soluble amyloid-β (Aβ) oligomer accumulation in the cortex and hippocampus in 5xFAD mouse offspring, indicating that antenatal hypoxia increased the vulnerability of the brain to synaptotoxic Aβ in the disease onset later in life. Consistent with the early occurrence of cognitive decline, we found synapse loss but not neuronal death in the cerebral cortex in 5xFAD but not wild-type (WT) offspring exposed to antenatal hypoxia. Furthermore, we also demonstrated that antenatal hypoxia significantly increased microglial number and activation, and reactive astrogliosis in the cerebral cortex in WT offspring. Moreover, antenatal hypoxia resulted in an exacerbated increase of microgliosis and astrogliosis in the early stage of AD in 5xFAD offspring. Together, our study reveals a causative link between fetal stress and the accelerated onset of AD-related pathology, and provides mechanistic insights into the developmental origin of aging-related neurodegenerative disorders.

## Introduction

Alzheimer’s disease (AD) is a progressive neurodegenerative disorder associated with cognitive impairment, memory loss, and later dementia among the elderly, which accounts for about 50–75% of all dementia cases (Hebert et al., 2013; Van Cauwenberghe et al., 2016; Jack et al., 2018). Currently, about 5 million Americans suffer from AD and related dementia (ADRD), and it is predicted to increase to 13.8 million in 2050 (Hebert et al., 2013). According to the “amyloid hypothesis,” the accumulation of soluble amyloid-β protein (Aβ) in the brain is one of the key events driving cognitive dysfunction and later dementia (Rosenblum, 1997; Hardy and Selkoe, 2002; Zlokovic, 2013; Selkoe and Hardy, 2016; Hecht et al., 2018). In addition to genetic factors, environmental factors also contribute to the onset and progression of AD pathology (Herrup, 2010; Braskie et al., 2011; Bakulski et al., 2012; Killin et al., 2016). Growing evidence indicates that AD/ADRD is a chronic neurological disorder throughout the lifespan with critical influences starting at conception and early life (Moceri et al., 2000; Seifan et al., 2015; Lemche, 2018). Large-scale human epidemiological studies have suggested that stress during fetal development and/or in early life may promote cognitive decline and accelerate the onset of AD-like pathology later in life (Wang et al., 2017; Lesuis et al., 2018). Recent animal studies also have shown that early life adversity affect AD-like neuropathology, such as Aβ levels and cognition functions at later ages (Sierksma et al., 2013; Zhang et al., 2013; Lesuis et al., 2017).

The “fetal origins of adult disease” hypothesis (Barker, 2004; de Boo and Harding, 2006; Langley-Evans and McMullen, 2010; Warner and Ozanne, 2010) has revealed that adverse prenatal environment can change developmental trajectories of tissue/organs in early life and increase the risk of diseases later in life, including heart diseases, and metabolic and neurobehavioral disorders (Barker et al., 1993, 2009; Gluckman and Hanson, 2004; Gluckman et al., 2008; Harris and Seckl, 2011). Hypoxia during gestation is a common form of fetal stress, which causes fetal growth restriction (low birth weight) and vital systemic developmental malformations, particularly the brain (Katz et al., 2001; Miller et al., 2016; Phillips et al., 2017). Not all developmental abnormalities caused by fetal stress may present apparent pathological phenotype in late life until “second hit” occurs (Basha et al., 2005; Lahiri et al., 2009). Our previous studies have demonstrated that adverse environmental factors during fetal development, including hypoxia, increase the susceptibility of the brain to hypoxic/ischemic insult in offspring (Tong et al., 2010; Ma et al., 2014; Li et al., 2016, 2017, 2019). However, whether and to what extent fetal stress influences the onset of AD-related pathology remain unexplored.

In the present study, we aim to determine the link between antenatal hypoxia and the onset of AD-like pathology later in life. Using an optimized animal model of hypoxia on pregnant dams, we find that antenatal hypoxia causes a significant reduction of brain and body weight in the fetal and the early postnatal period in mouse offspring. Most notably, we demonstrate that antenatal hypoxia accelerates the occurrence of cognitive decline in offspring of 5xFAD mice. This early occurrence of cognitive impairment is associated with increased synapse loss and augmented microglial number and activation and reactive astrocytes in the cerebral cortex in offspring of 5xFAD mice. Moreover, antenatal hypoxia has no significant effect on the accumulation of soluble Aβ oligomers in the cortex and hippocampus in the early stage of AD in 5xFAD offspring. Thus, we demonstrate a link between fetal stress and an acceleration in the development of AD pathology later in life, and suggest a novel mechanism of increased susceptibility of the brain to soluble Aβ accumulation as a risk factor to modulate the onset of ADRD.

## Materials and Methods

### Animals

Male 5xFAD (JAX MMRRC Stock #034848) and female wild-type (WT) mice with a C57BL/6J background were purchased from The Jackson Laboratory. The mouse colonies were maintained by crossing male hemizygous transgenic mice with female C57BL/6J mice. All mice were group housed after mating and body weights were taken every week. Pregnancy was noticed by significant weight gain at 14 days after mating. The pups were kept with the dam in single cage and group housed after weaning at 21 days after birth. All mice were bred in the Animal Care Facility of Loma Linda University, had access to an open-formula diet (LabDiet 5LG4) and water *ad libitum*, and were housed under a 12-h light–dark cycle at 22–24°C. All procedures and protocol were approved by the Institutional Animal Care and Use Committee of Loma Linda University and followed the guidelines by the National Institutes of Health Guide for the Care and Use of Laboratory Animals.

### Antenatal Hypoxia Treatment

Pregnant C57BL/6J mice were randomly divided into two groups to receive either normoxia or hypoxia treatment during gestation. To induce hypoxic responses, the animals were placed in their home cages in a hypoxia chamber with 11.5 or 10.5% O_2_ generated by an Altitude Generator (Everest Summit II; Hypoxico Altitude Training System, New York, NY, USA) from days 14.5 to 17.5 of gestation (E14.5–E17.5), as described previously (Gortner et al., 2005; Tomlinson et al., 2010; Meng et al., 2020). At the end of exposure, the dams were returned to normoxic conditions. Oxygen in the chamber was measured using an oxygen analyzer (OxyCheq Expedition-X, FL, USA). The normoxia control was housed identically with room air. Some mice were euthanized on day 18.5 of pregnancy (E18.5), and fetuses and fetal brains were collected for analysis. Other mice were allowed to give birth, and further studies were conducted at postnatal day 3 (P3), 21 (P21), 2 months, and 4 months of both sexes. The pups of pregnant mice were genotyped by 2 weeks after birth using standard polymerase chain reaction (PCR) according to the genotyping protocol of The Jackson Laboratory.

### Neurobehavioral Test

The following neurobehavioral tests were performed in mouse offspring and were recorded for further analysis. Spontaneous Y-maze (sp-Y) test was used to evaluate the spontaneous alternation performance as described previously (Ohno et al., 2004; Oakley et al., 2006). The animal was introduced to one arm of Y-shaped maze with three plastic arms at a 120° angles from each other and was allowed to freely explore the three arms during a 5-min session. The number of arm entries and the number of triads were counted and the percentage of alternation was calculated as follows: number of triads containing entries into all three arms/maximum possible alternations (the total number of arms entered − 2) × 100. The open field (OF) test (Li et al., 2016) and novel object recognition (NOR) test (Wolf et al., 2016) were conducted in a square test box (40 × 40 × 40 cm^3^). Each mouse was placed in the arena and allowed to freely explore the box for 10 min, which was recorded by an overhead cam. Distance moved, time spent, and entries in center were calculated by analyzing the recorded video. The NOR test includes three trials conducted in 3 days. A habituation trial was conducted on day 1, the same as OF test. Twenty-four hours after habituation, two identical sample objects were introduced into the box. The mouse was allowed to freely explore objects for 10 min and then returned to its home cage. The test trial was conducted 24 h later and involved the introduction of one novel object and one familiar sample object at the same positions as day 2. The animal was exposed to these two objects for 10 min. The interaction of the mouse with the familiar object or novel object was videotaped, and the time spent exploring each object was recorded by using timers. The discrimination index (DI) was calculated as follows: (time spent on the novel object–time spent on the familiar object)/total exploration time. The data of sp-Y test and OF test were analyzed using NIH imageJ software with MouBeAT plugin (Wolf et al., 2016).

### qRT-PCR

Total RNA was isolated from cerebral cortex of the mouse brain hemisphere at 2 months of age using Trizol reagent (15596018, Ambion). Total RNA was subjected to reverse transcription with Superscript III First-Strand Synthesis System (18080051; Invitrogen), following the manufacturer’s instructions. The mRNA abundance was determined with real-time PCR using iQ SYBR Green Supermix (1708880; Bio-Rad) as we previously described (Ma et al., 2016). Real-time PCR was performed in a final volume of 25 μl and each PCR reaction mixture consisted of specific primers (Table 1) and iQ SYBR Green Supermix. PCR was done in triplicate and threshold cycle numbers were averaged for each sample. The expression of Iba1, Gfap, Cd68, and Tmem119 were assessed using the primer sequences listed in Table 1. The relative expression levels were calculated using the formula 2(^−ΔΔCt^) and normalized to actin. The changes of target transcripts were expressed as fold of normal control.

**Table 1:** qRT-PCR primers.

Gene	Forward primer (5′ → 3′)	Reverse primer (5′ → 3′)
*Iba1*	GTCCTTGAAGCGAATGCTGG	CATTCTCAAGATGGCAGATC
*Gfap*	TCCTGGAACAGCAAAACAAG	CAGCCTCAGGTTGGTTTCAT
*Cx3cr1*	CAGCATCGACCGGTACCTT	GCTGCACTGTCCGGTTGTT
*Cd11b*	CCTTCATCAACACAACCAGAGTGG	CGAGGTGCTCCTAAAACCAAGC
*Cd68*	TTCACCTTGACCTGCTCTCTC	GTAGGTTGATTGTCGTCTGCG
*Tmem119*	CCTTCACCCAGAGCTGGTTC	GGCTACATCCTCCAGGAAGG

### Western Blotting

Total protein was extracted from cerebral cortex of brain hemisphere. Briefly, brain tissue was homogenized in RIPA lysis buffer (CST) with further centrifugation at 14,000× *g* at 4°C for 30 min. Protein concentration in the supernatant was determined using the BCA assay kit (Bio-Rad). Equal amounts of protein were loaded on a SDS–PAGE gel. After being electrophoresed and transferred to a PVDF membrane, the membrane was blocked and incubated with the primary antibodies against Synapsin 1 (CST; 1:2,000), Post-synaptic density protein 95 (PSD95; Thermo Fisher Scientific, Waltham, MA, USA; 1:2,000), Aβ (CST; 1:2,000), actin (Sigma; 1:6,000), or GAPDH (Abcam; 1:5,000) overnight at 4°C. The membrane was then incubated with fluorescence-labeled secondary antibodies (Thermo Fisher Scientific) for 1 h at room temperature against light. After washing, the membrane was then scanned with the Odyssey CLx Imaging System. The data were analyzed with the NIH ImageJ software. The values in the figures represent relative density of the bands normalized to GAPDH/actin.

### Immunofluorescence Staining and Confocal Microscopy

Mouse pups were transcardially perfused with ice-cold heparinized PBS followed by 4% PFA. The brains were post-fixed in 4% PFA overnight, dehydrated with 30% sucrose solution in PBS at 4°C, and allowed to completely sink to the bottom of the container. Then the dehydrated brains were dissected, embedded into Tissue-Tek optimal cutting temperature compound (OCT; VWR) on dry ice, and cryosectioned at a thickness of 20 μm for immunofluorescence staining. Brain sections from 1 mm anterior to Bregma to 3 mm posterior to Bregma were collected. Slices were blocked with 5% donkey serum (Jackson ImmunoResearch) containing 0.3% Triton X-100 (Sigma–Aldrich) at room temperature for 1 h, and then incubated with following primary antibodies: NeuN (Abcam; 1:1,000), Iba1 (Wako; 1:500), CD68 (Bio-Rad; 1:500), Synapsin 1 (CST; 1:400), Aβ (CST; 1:1,000), or GFAP (Abcam; 1:1,000), overnight at 4°C. After washing, slices were incubated with Alexa Fluor 594- or 488-conjugated secondary antibodies (Invitrogen, 1:500) for 1 h at room temperature. The cell nuclei was stained by DAPI for 15 min after incubating with secondary antibodies. Cell death was detected by TUNEL staining using the *in situ* Cell Death Detection Kit, Fluorescein (Roche) following neuron (NeuN) staining. The TUNEL staining was completed according to the manufacturer’s instructions. Tissue slices were mounted with fluorescent mounting media (Dako) and covered with coverslips. All slides were scanned with a Zeiss LSM 710 NLO confocal microscope (Zeiss) in the Advanced Imaging and Microscopy Facility of Center of Perinatal Biology. Images of the hippocampus and the cerebral cortex were captured and analyzed using the NIH ImageJ software.

### Statistical Analysis

Data were expressed as mean ± SEM. All graphs in this study were generated with GraphPad Prism 5. In experiments related to animals, experimental number (*n*) represents mouse offspring from at least two different dams. Comparisons between two groups were analyzed using Student’s *t*-test (unpaired, two-tailed), and multiple comparisons were analyzed using one-way ANOVA followed by Newman–Keuls *post hoc* test. A *p*-value <0.05 was considered significant. Linear regression and Pearson correlation were used to determine the correlation between data sets using GraphPad Prism.

## Results

### Antenatal Hypoxia Caused Growth Restriction in Offspring

**Figure 1:**
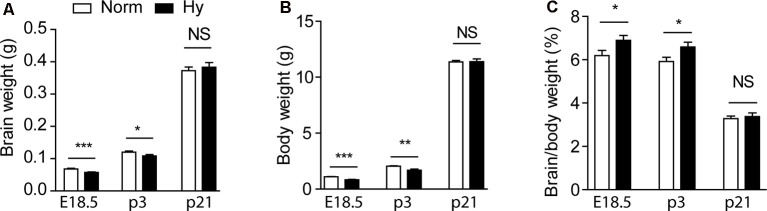
Antenatal hypoxia caused growth restriction in 5xFAD offspring. Pregnant mice were treated with normoxia or hypoxia from day 14.5 to day 17.5 of gestation. Brain weight **(A)**, body weight **(B)**, and the ratio of brain to body weight **(C)** of fetuses at embryonic day 18.5 (E18.5), and offspring at postnatal day 3 (P3) and 21 (P21) were evaluated. Data are mean ± SEM. Student’s *t*-test was applied to each data set. ***p < 0.0001; **p < 0.001; *p < 0.05; NS, not significant. E18.5, *n* = 17–18; P3, *n* = 11–14; P21, *n* = 6.

First, we investigated the impact of antenatal hypoxia on the normal growth of fetuses and pups. To optimize the oxygen concentration of hypoxic treatment, pregnant dams were treated with normoxia or two different hypoxia conditions (11.5 or 10.5%) from gestational day 14.5 (E14.5) to 17.5 (E17.5) for 3 days. Antenatal hypoxia of 11.5% resulted in a significant reduction in brain weight by about 16% (Figure 1A, *p* < 0.0001) and body weight by about 24% (Figure 1B, *p* < 0.0001) in E18.5 fetuses, compared with normoxia control. There remained about 10% reduction in brain weight (Figure 1A, *p* < 0.05) and about 18% reduction in body weight (Figure 1B, *p* < 0.001) in postnatal day 3 (P3) pups of hypoxic treated dams. At P21, no significant differences were observed in either brain or body weight between normoxia and hypoxia groups (Figures 1A,B, NS), suggesting a catch-up growth after birth in offspring of hypoxic treated dams. In addition, antenatal hypoxia treatment did not affect the body weight of male or female 5xFAD offspring at 2 months, compared with normoxia control (Supplementary Figures S1B,C). We also observed the brain-sparing effect in response to fetal hypoxia, showing a significant increase in the brain to body weight ratio in E18.5 fetuses and P3 pups (Figure 1C, *p* < 0.05), but not in P21 animals (Figure 1C, NS). In consistence with previous studies, our results also showed that fetal hypoxia did not significantly affect litter size, compared with the normoxia control (Norm: 5.417 ± 0.468; Hy: 6.182 ± 0.377, *p* = 0.22; Supplementary Figure S1A). In comparison with 11.5% O_2_ treatment, antenatal hypoxia with 10.5% O_2_ not only caused more severe reduction in brain weight (about 19% in P3 pups) and body weight (about 26% in P3 pups; Supplementary Figures S1D–F) but also induced more pregnancy loss. Thus, antenatal hypoxia with 11.5% was conducted to further evaluate that onset of AD-related pathology in 5xFAD offspring.

### Antenatal Hypoxia Exacerbated Cognitive Impairment in 5xFAD Offspring

**Figure 2:**
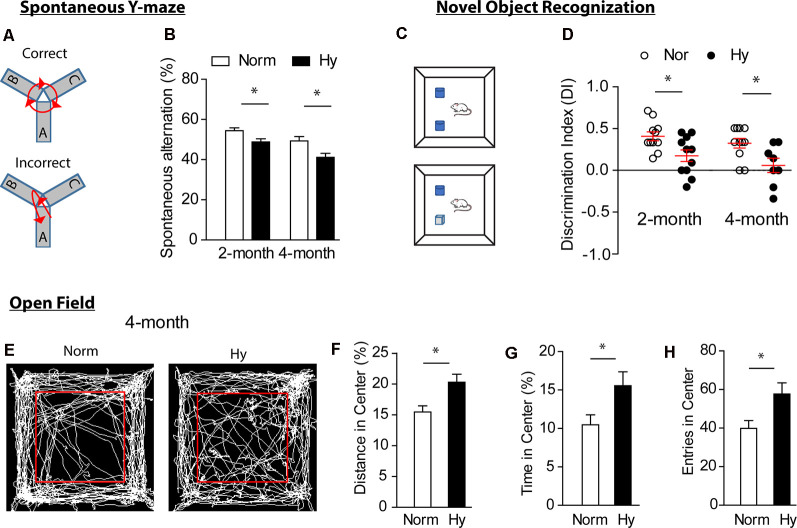
Antenatal hypoxia exacerbated cognitive impairment in 5xFAD offspring. Spontaneous Y-maze test (sp-Y, **A,B**) and novel object recognition (NOR, **C,D**) were conducted at 2 and 4 months. **(A)** Schematic of the sp-Y test. **(B)** Spontaneous alternation of 5xFAD offspring exposed to antenatal hypoxia or normoxia. **(C)** Schematic of the NOR test. **(D)** Discrimination index (DI) of 5xFAD offspring exposed to antenatal hypoxia or normoxia. Open field test (OF, **E–H**) was performed at 4 months. **(E)** Schematic of the OF test and movement tracks from normoxia- or hypoxia-treated 5xFAD offspring. The red square separates inner zone (center) and outer zone. **(F)** Distance in center, **(G)** time in center, and **(H)** entries in center of offspring exposed to antenatal hypoxia or normoxia. Data are mean ± SEM. Student’s *t*-test was applied to each data set. *p < 0.05. Sp-Y test at 2 months: Norm, *n* = 15; Hy, *n* = 12. Sp-Y test at 4 months: Norm, *n* = 15; Hy, *n* = 7. NOR at 2 months: *n* = 11. NOR at 4 months: Norm, *n* = 12; Hy, *n* = 8. OF: Norm, *n* = 12, Hy, *n* = 8.

Next, we investigated whether antenatal hypoxia exacerbates cognitive impairment in 5xFAD mouse offspring. A battery of neurobehavioral tests for cognitive functions, including sp-Y test (Oakley et al., 2006; Wolf et al., 2016), NOR test, and OF test (Wolf et al., 2016), were conducted in offspring of 5xFAD treated with either normoxia or hypoxia during fetal development. The sp-Y test measures the spatial working memory of offspring (Figures 2A,B). The results showed that antenatal hypoxia significantly reduced spontaneous alternation from 54.4 to 48.8% at 2 months (Figure 2B, *p* < 0.05) and from 50.1 to 41.8% at 4 months (Figure 2B, *p* < 0.05), compared with normoxia control. In addition, no significant differences were observed in total entries and total travel distance in Y-maze test in offspring at 2 and 4 months between normoxia and hypoxia groups (Supplementary Figure S2). These data indicate that fetal hypoxia did not affect the initiation of motor deficits, which were observed in the late stage of AD in 5xFAD mice (O’Leary et al., 2018; Creighton et al., 2019).

We then evaluated the exploration and discrimination abilities in offspring with the NOR test (Figures 2C,D). The preference of animals to novel object was present as DI. The result showed that offspring exposed to antenatal hypoxia spent less time on novel object than old object, showing a significantly reduced DI value at both 2 and 4 months (Figure 2D), compared with normoxia control. The OF test was performed to assess the impact of fetal hypoxia on the development of anxiety in 5xFAD offspring at 4 months (Figures 2E–H). Interestingly, 5xFAD offspring of antenatal hypoxia traveled a greater distance (Figure 2H) and spent more time in the center (Figure 2G) than those from the normoxia control, suggesting a change in the anxiety level, which is usually observed in 12-month-old 5xFAD mice (Creighton et al., 2019). The total entries in the center were also significantly increased in 5xFAD offspring exposed to antenatal hypoxia, as compared with normoxia control (Figure 2H).

### Antenatal Hypoxia Did Not Affect Soluble Aβ Levels in the Early Stage of AD in 5xFAD Offspring

**Figure 3:**
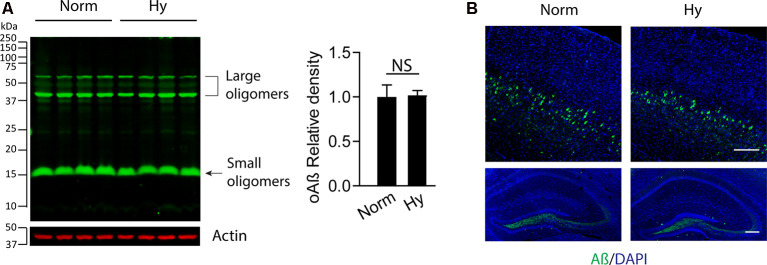
Antenatal hypoxia did not affect soluble Aβ accumulation in the brain cortex and hippocampus of 2-month-old 5xFAD offspring. The brain cortex was separated from the brain of 2-month-old 5xFAD offspring exposed to normoxia or hypoxia. **(A)** Western blotting was performed to detect the soluble Aβ levels with primary antibody against soluble Aβ (oAβ). Actin was used as internal control. Data are mean ± SEM. Student’s *t* test was applied to each data set. NS, not significant, *n* = 4. **(B)** Confocal imaging of soluble Aβ (green) distribution in the cortex (upper panel) and hippocampus (lower panel) in the brain of 2-month-old 5xFAD offspring exposed to normoxia or hypoxia. DAPI stains nuclei (blue). Scale bar: 200 μm.

To test the potential effect of antenatal hypoxia on Aβ accumulation in the cortex in the early stage of AD in 5xFAD mice, we separated the brain cortex from 2-month-old 5xFAD offspring exposed to normoxia or antenatal hypoxia. The Western blot result showed that there was no significant difference of soluble Aβ (oAβ) levels in the cortex in 2-month-old 5xFAD offspring between normoxia or hypoxia groups (Figure 3A). We also performed immunofluorescence staining to visualize the distribution of soluble Aβ in the brain. The confocal images showed that the intraneuronal soluble Aβ (green) was the predominant form in the brain of 2-month-old 5xFAD offspring, which distributed in the cortical deep layers with somewhat lower expression in the dentate gyrus region of hippocampus (Figure 3B). Thus, our results suggest that antenatal hypoxia increased the vulnerability of the brain to the accumulation of soluble Aβ in offspring.

### Antenatal Hypoxia Potentiated Synapse Loss in 5xFAD Offspring

**Figure 4:**
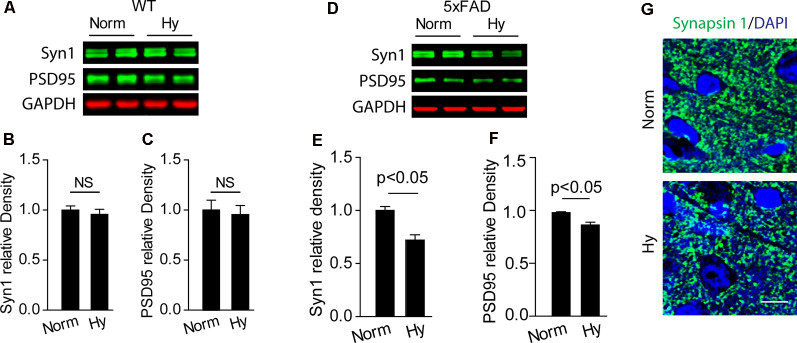
Antenatal hypoxia resulted in synaptic loss in the brain cortex of 2-month-old 5xFAD offspring. **(A–C)** Western blotting analysis of the presynaptic marker Syn1 and postsynaptic marker PSD95 in the cortex of 2-month-old wild-type (WT) and **(D–F)** 5xFAD mice exposed to normoxia or hypoxia. Actin was used as internal control. Data are mean ± SEM. Student’s *t*-test was applied to each data set. *n* = 3–4. **(G)** Confocal images of brain slices from 2-month-old 5xFAD offspring exposed to normoxia or hypoxia stained with antibody against Synapsin 1 (green). DAPI stains nuclei (blue). Scale bar: 10 μm. NS, not significant.

We measured the expression of synaptic makers in the cerebral cortex of 2-month-old offspring of 5xFAD exposed to antenatal hypoxia or normoxia. Our results showed that antenatal hypoxia resulted in significant reduction of Synapsin 1 (Syn1) by about 28% (Figures 4D,E, *p* < 0.05) and PSD95 by about 12% (Figures 4D,F, *p* < 0.05) in 5xFAD mice, but not in WT mice (Figures 4A–C). The confocal images also showed the reduction of Syn1 (green) density in the cortex of offspring exposed to fetal hypoxia, compared with normoxia control (Figure 4G). In addition, TUNEL staining was performed on brain slices to determine the potential neuronal loss. The confocal microscopy analysis demonstrated no differences in TUNEL-positive (red) neuronal (NeuN, green) death in the brain cortex of 5xFAD offspring between normoxia and hypoxia groups (Supplementary Figure S3).

### Antenatal Hypoxia Increased Microglial Number and Activation and Reactive Astrogliosis in 5xFAD Offspring

**Figure 5:**
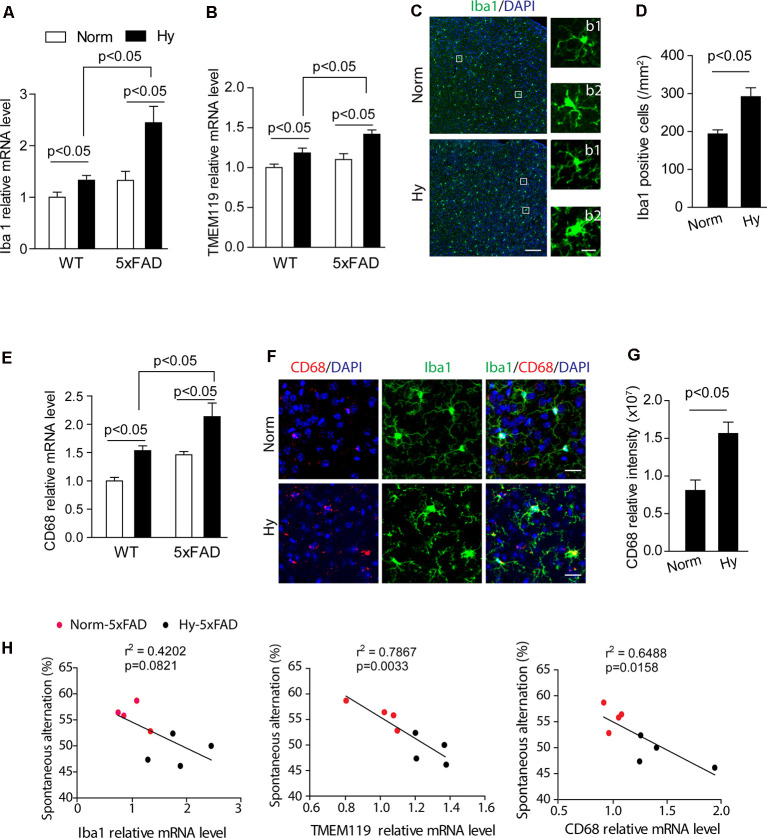
Antenatal hypoxia increased microglial activation in the brain cortex of 2-month-old 5xFAD offspring. **(A,B)** qRT-PCR of mRNA levels of microglial markers Iba1, TMEM119 in the cortex of WT and 5xFAD offspring with antenatal hypoxia or normoxia treatment. Data are mean ± SEM. Student’s *t*-test was applied to each data set. *n* = 4. **(C)** Confocal images of coronal brain slices of 2-month-old 5xFAD offspring with antenatal hypoxia or normoxia treatment stained with antibody against Iba1 (green). DAPI strains nuclei (blue). Insert **(b1)** shows the resting microglia and **(b2)** shows the activated microglia. Scale bar: **(B)**, 100 μm; **(b1, b2)**, 20 μm. **(D)** Quantification of Iba1-positive cell numbers in the cerebral cortex with ×20 objective. Student’s *t*-test was applied to each data set. A total of nine sections from three animals were used for analysis. **(E)** qRT-PCR of mRNA levels of the activated microglia marker CD68 in the cortex of the WT and 5xFAD mice exposed to antenatal hypoxia and normoxia. Data are mean ± SEM. Student’s *t*-test was applied to each data set. *n* = 4. **(F)** Confocal images of coronal brain slices of 2-month-old 5xFAD mouse offspring with antenatal hypoxia or normoxia treatment stained with antibody against CD68 (red) an Iba1 (green). DAPI stains nuclei (blue). **(G)** Quantification of CD68 fluorescence intensity in the cerebral cortex with ×20 objective. Data are mean ± SEM. Student’s *t*-test was applied to each data set. A total of nine sections from three animals were used for analysis. Scale bar: 20 μm. **(H)** Regression analysis of the correlation between spontaneous Y-maze performance with relative mRNA level of Iba1, TMEM119, and CD68, respectively. Individual data points are from 5xFAD normoxia (red) and hypoxia treated mice (blue).

To study the effect of antenatal hypoxia on microgliosis, we measured the effect of antenatal hypoxia on the expression of multiple specific brain microglial markers Iba1 and TMEM119 in the brain cortex of 2-month-old 5xFAD offspring using qRT-PCR. The results showed that in 2-month-old WT and 5xFAD offspring, antenatal hypoxia significantly increased the expression of microglial markers Iba1 and TMEM119 compared with normoxia control (Figures 5A,B, *p* < 0.05). Moreover, with antenatal hypoxia treatment, 5xFAD offspring exhibited more remarkable upregulation of microglial markers than WT offspring (*p* < 0.05). Activated microglia can change their ramified morphology to shorter, thick processes and rod-shape morphology (Nayak et al., 2014; Hong et al., 2016). Using confocal microscopy to visualize microglial morphological alternations (Figure 5C), we found that both resting (Figures 5C,b1) and activated (Figures 5C,b2) microglia were observed in the brain cortex of 5xFAD offspring exposed to normoxia or antenatal hypoxia. The subsequent quantification of Iba1-positive signal revealed that antenatal hypoxia significantly increased Iba1-positive cell number by about 51% in the brain cortex of 5xFAD offspring, compared with normoxia control (Figure 5D, *p* < 0.05). To further confirm microglial activation, we measured the expression of microglial activation and phagocytic markers CD68 using qRT-PCR. The results showed that fetal hypoxia significantly upregulated the mRNA levels of CD68 in the cortex of WT or 5xFAD offspring at 2 months (Figure 5E, *p* < 0.05), compared with normoxia control. Moreover, with antenatal hypoxia treatment, 5xFAD offspring exhibited more remarkable upregulation of CD68 than WT offspring (*p* < 0.05). Using double immunostaining of CD68 (red) and Iba1 (green) on brain slices to visualize microglial activation, we observed enhanced CD68 signals in Iba1-positive cells in the brain cortex of 2-month-old 5xFAD offspring of antenatal hypoxia (Figure 5F), compared with normoxia control. Moreover, the subsequent quantitative results demonstrated that antenatal hypoxia resulted in a significant increase of CD68 signal intensity by about 94% (Figure 5G, *p* < 0.05) in the brain cortex of 2-month-old 5xFAD offspring, compared with normoxia control. A negative correlation was found between spontaneous alteration and the expression of Iba1, TMEM119, and CD68 (Figure 5H). The greater the increase in Iba1, TMEM119, or CD68 expression, the mice are less likely to alternate between arms, indicating a deficiency in their cognitive ability.

**Figure 6:**
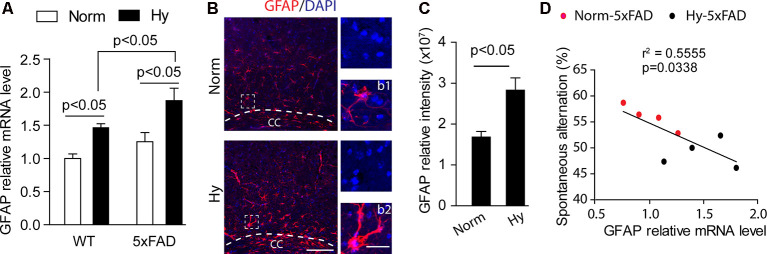
Antenatal hypoxia increased reactive astrogliosis in the brain cortex of 2-month-old 5xFAD offspring. **(A)** qRT-PCR of GFAP mRNA levels in the cortex of offspring exposed to normoxia or hypoxia. Student’s *t*-test was applied to each data set. *n* = 4. **(B)** Confocal images of brain slices from 2-month-old 5xFAD offspring exposed to normoxia or hypoxia stained with antibody against GFAP (red). DAPI stains nuclei (blue). Insert **(b1)** shows normal astrocyte and **(b2)** shows the reactive astrocyte. Scale bar: **(B)**, 100 μm; **(b1,b2)**, 20 μm. **(C)** Quantification of GFAP fluorescence intensity in the cerebral cortex with ×20 objective. Data are mean ± SEM. Student’s *t*-test was applied to each data set. In total, nine sections from three animals were used for analysis. **(D)** Regression analysis of the correlation between spontaneous Y-maze performance with GFAP relative mRNA level. Single data points are from 5xFAD normoxia (red) and hypoxia treated mice (blue).

Astrocytes are a type of glia cells abundantly distributed in the central nervous system, which supply nutrients for neural development, maintain the brain environment, and provide brain defense (Wang and Bordey, 2008). Our results showed that in 2-month-old WT and 5xFAD offspring, antenatal hypoxia significantly increased the mRNA level of astrocyte marker GFAP, compared with normoxia control (Figure 6A, *p* < 0.05). Moreover, with antenatal hypoxia treatment, 5xFAD offspring exhibited more remarkable upregulation of GFAP than WT offspring (*p* < 0.05). Using immunostaining of GFAP on brain slices of 2-month-old 5xFAD offspring, we observed an increase in GFAP-positive staining (red) with reactive astrogliosis morphology in the brain cortex, especially the region above the lateral corpus callosum (CC; Figure 6B) in offspring exposure to antenatal hypoxia. The reactive astrocytes in the hypoxic group showed enlarged cell body and longer astrocyte processes (Figures 6B,b2), compared with resting astrocytes (Figures 6B,b1) seen in normoxia group. The subsequent quantification of GFAP-positive signals revealed that antenatal hypoxia significantly increased GFAP-positive signal intensity in the brain cortex by about 68% in 5xFAD offspring, compared with normoxia control (Figure 6C, *p* < 0.05). A negative correlation was found between the GFAP expression and spontaneous alternation. Higher GFAP expression is related to less spontaneous alternation the Y maze (Figure 6D).

## Discussion

Our previous studies have demonstrated that antenatal hypoxia induces low brain and body weight and shows a detrimental effect on neuronal survivability in offspring of rats (Tong et al., 2010; Gonzalez-Rodriguez et al., 2014). In the present study, we have firstly established the causative link between antenatal hypoxia and the onset of AD-related dementia, and recapitulated major aspects of AD pathology in the early stage of AD in 5xFAD offspring. Emerging evidence suggested that early life adversity was associated with the onset and progression of AD/ADRD pathogenesis later in life (Moceri et al., 2000; Seifan et al., 2015; Lemche, 2018). Human epidemiological studies have shown that infants of low birth weight or small head circumference for gestational age have a significantly greater risk of age-related cognitive impairment or dementia in late adulthood, compared with those with normal growth (Skogen et al., 2013; Heinonen et al., 2015; Mosing et al., 2018). Small birth size has been considered as an indicator of an adverse intrauterine environment, thus this evidence indicates that early-life stress experience, next to genetic causes, is a risk factor to modulate the development and progression of AD/ADRD. To test this hypothesis, we applied antenatal hypoxia treatment on pregnant dams, which has been widely used in studying fetal stress-induced growth restriction on the programming of brain and heart diseases later in life (Li et al., 2012, 2017; Ma et al., 2014; Xiong et al., 2016; Martinez et al., 2017; Lv et al., 2019). In consistence with previous findings, our results showed that antenatal hypoxia induced fetal growth restriction in the fetal and the early postnatal period in mice, showing significantly reduced brain and body weight, which recovered in young adult offspring in both genders as a result of catch-up growth. In addition, our antenatal hypoxia paradigm with 11.5% O_2_ treatment did not affect the litter size. These results suggest a suitable model of fetal stress in the study of onset and progression of AD-related pathology later in life.

AD is clinically characterized by age-related cognitive decline and behavioral symptoms (Selkoe and Schenk, 2003). Our study used 5xFAD transgenic mouse model of AD, which expresses human amyloid precursor protein (APP) and presenilin-1 (PS1) transgenes with five AD-linked mutations, and rapidly develops AD-related neuropathology and cognitive dysfunctions (Oakley et al., 2006). To evaluate the impact of antenatal hypoxia on the development of cognitive impairment later in life, we performed spontaneous Y-maze, novel object, and OF tests to examine cognitive deficits in 5xFAD offspring. We found that antenatal hypoxia induced cognitive decline as early as 2 months in 5xFAD offspring, which usually occurred 4–6 months later in 5xFAD mice in previous studies (Oakley et al., 2006; Jawhar et al., 2012). Thus, our data suggest that antenatal hypoxia accelerates the occurrence of cognitive decline in 5xFAD offspring. The accumulation of soluble Aβ oligomers rather than amyloid deposits in the brain is the primary cause of cognitive impairment of AD (Dahlgren et al., 2002; Hardy and Selkoe, 2002; Gandy, 2005), which has been demonstrated in 5xFAD mice (Ohno et al., 2004; Oakley et al., 2006). The accumulation of soluble Aβ occurs in the cortex layer V at 1.5–2 months of age in 5xFAD mice (Oakley et al., 2006; Eimer and Vassar, 2013). Using an antibody detecting Aβ oligomers (Ma et al., 2018), we found that soluble Aβ oligomers were predominant species in both cortex and hippocampus in 2-month-old 5xFAD offspring, which were not significantly changed by either antenatal hypoxia or normoxia, thus suggesting that fetal hypoxia reprograms the normal brain development trajectory leading to an increase in vulnerability of the brain to synaptotoxic Aβ accumulation in the early stage of AD pathogenesis.

It has been demonstrated that synaptic loss strongly correlates with cognitive impairment in AD (DeKosky and Scheff, 1990; Terry et al., 1991; Hong et al., 2016). In correlation with early onset of cognitive decline, we observed significant decrease of cortical synapse markers Syn1 and PSD95 in 2-month-old 5xFAD offspring exposed to antenatal hypoxia, which occurred in somatosensory and prefrontal cortices of 5xFAD mice at 6 months (Crowe and Ellis-Davies, 2014). Mounting evidence has shown that fetal stress including fetal hypoxia increases the risk of developing neuropsychiatric disorders and developmental disabilities later in life, which is related to maternal stress-induced synapse loss (Hayashi et al., 1998; Giannopoulou et al., 2018). Our previous studies also demonstrated that antenatal hypoxia affected normal brain development (Tong et al., 2010) and promoted the development of hypoxia/ischemia-sensitive phenotype in the brain of rat pups (Li et al., 2012, 2016; Gonzalez-Rodriguez et al., 2014). In addition, fetal hypoxia is associated with altered cerebral cortical structure, increased microglial activation and astrogliosis, and reduced myelinated fibers (Pham et al., 2015; Miller et al., 2016; Cahill et al., 2019). Moreover, in the present study, antenatal hypoxia treatment was conducted during embryonic days 14.5–17.5, which is a critical period of brain development including neurogenesis, synaptogenesis, initiation of synapse pruning, and microglia invasion into the cortex (Reemst et al., 2016). Thus, all the evidence suggests that antenatal hypoxia results in brain abnormal development and is associated with early synapse loss in 5xFAD offspring.

Gliosis also contributes to early synapse loss and cognitive impairment in the early stage of AD pathogenesis (Hong et al., 2016). Microgliosis and astrogliosis are observed in the brain of 5xFAD mice at 2 months (Oakley et al., 2006; Giannoni et al., 2016). Reactive astrogliosis is commonly observed in the progression of Aβ pathology and is an early hallmark of AD in humans and mouse models (Osborn et al., 2016; Reichenbach et al., 2019). Moreover, it has been reported that activated microglia can induce neurotoxic reactive astrocytes (Liddelow et al., 2017). Our study demonstrated increased reactive microgliosis and astrogliosis in 2-month-old 5xFAD offspring exposed to antenatal hypoxia. Microglia play a crucial mediating role in synaptic refinement during the development of neuronal connections through elimination of synapse elements (Stevens et al., 2007; Paolicelli et al., 2011; Schafer et al., 2012; Bialas and Stevens, 2013). The perinatal period is a critical window for microglial migration, proliferation, and plasticity, and therefore is particularly vulnerable to environmental influences that may induce long-term changes in microglial cell number and/or phenotypes (Hanamsagar and Bilbo, 2017). Moreover, it has been reported that prenatal stress causes priming of microglial activation (Diz-Chaves et al., 2012; Ślusarczyk et al., 2015; Desplats et al., 2019), which increases the sensitivity of microglia facing the second challenge in adulthood (Perry and Holmes, 2014; Desplats et al., 2019). Microglia can be activated by Aβ accumulation and represent a common pathological feature of AD (Sarlus and Heneka, 2017). They are also critically involved in the early steps of AD pathology (Hong et al., 2016; Hemonnot et al., 2019). Aberrant microglial activation by soluble Aβ oligomers mediates early synapse loss in AD mouse models in the early stage of AD-like pathology (Hong et al., 2016) and human AD brain (Wu et al., 2019). We observed elevated expression of a group of phagocytosis markers in the early stage of AD in 5xFAD mice exposed to antenatal hypoxia, which may be associated with early synapse loss and accelerated cognitive decline. Future studies should reveal whether and how antenatal hypoxia primes microglia functions during the brain development and contributes to synapse loss in 5xFAD offspring later in life.

## Conclusion

In summary, we find that antenatal hypoxia induces low brain and body weight in mouse fetuses and neonates, which is associated with the initiation of AD-related pathology. Moreover, 5xFAD offspring exposed to antenatal hypoxia exhibits early synaptic loss, intensive microglial activation, and reactive astrogliosis in the cerebral cortex in the early stage of AD, which provide support to the early occurrence of cognitive decline in 5xFAD offspring. Therefore, our study reveals a causative link between adverse maternal environments and the development of AD-related dementia, and provides insight into the fetal origin of aging-related neurodegenerative disorders.

## Data Availability Statement

All datasets presented in this study are included in the article/Supplementary Material.

## Ethics Statement

The animal study was reviewed and approved by Institutional Animal Care and Use Committee of Loma Linda University.

## Author Contributions

GS performed brain sample collection, western blotting, brain slicing, immunostaining, imaging and quantification. SH conducted animal maintenance, hypoxia treatment, behavioral test, sample collection, and RT-PCR. ZZ aided in experimental design and contributed to figure design. LZ contributed to article writing. QM conceived the study, interpreted data, and wrote the article. All authors contributed to the article and approved the submitted version.

## Conflict of Interest

The authors declare that the research was conducted in the absence of any commercial or financial relationships that could be construed as a potential conflict of interest.

## Abbreviations

Aβ: amyloid-β protein
AD: Alzheimer’s disease
ADRD: AD-related dementia
APP: amyloid precursor protein
NOR: novel object recognition
OF: open field
PS1: presenilin-1
PSD95: post-synaptic density protein 95
sp-Y: spontaneous Y-maze
Syn1: synapsin 1
WT: wild-type.
