# Supplementary material for: Antenatal Hypoxia Accelerates the Onset of Alzheimer’s Disease Pathology in 5xFAD Mouse Model
Source: Front Aging Neurosci. 2020 Aug 21;12:251. doi: 10.3389/fnagi.2020.00251 (PMC7472639; doi:10.3389/fnagi.2020.00251)
Supplement: Supplementary file 1 [file Table_1.DOCX]

**Supplementary**

**Figure S1**

**Effect of gestational hypoxia on growth restriction in 5xFAD offspring.** Pregnant dams were treated with normoxia or hypoxia (11.5%) from days 14.5 to days 17.5 of gestation. Litter size (A) was counted after birth. Body weight of female (B) and male (C) 5xFAD offspring was measured at 2 months. Pregnant mice were treated with normoxia or hypoxia (10.5%) from days 14.5 to days 17.5 of gestation. Brain weight (D), body weight (E) and brain to body weight ratio of P3 offspring were measured. Data are mean ± SEM. A Student’s *t*-test was applied to each data set. ***, p<0.0001, **, p<0.001, *, p<0.05. NS, no significant.

**Figure S2**

**Effect of gestational hypoxia on motor functions in 5xFAD offspring by Spontaneous Y-maze (sp-Y) test.** (A) total entries and (B) total distance at 2 months, and (C) total entries and (D) total distance at 4 months were measured in 5xFAD offspring by sp-Y test. Data are mean ± SEM. A Student’s *t*-test was applied to each data set. NS, no significant. NS, no significant. 2 months: Norm, n=15; Hy, n=12. 4 months: Norm, n=15; Hy, n=8.

**Figure S3**

**The effect of gestational hypoxia on neuronal loss in the brain cortex of 5xFAD offspring.** Confocal images of brain slices of 2-month-old 5xFAD offspring with antenatal hypoxia (Hy) or normoxia (Norm) treatment stained with TUNEL (red) colocalized with NeuN (green). DAPI stains nuclei (blue).


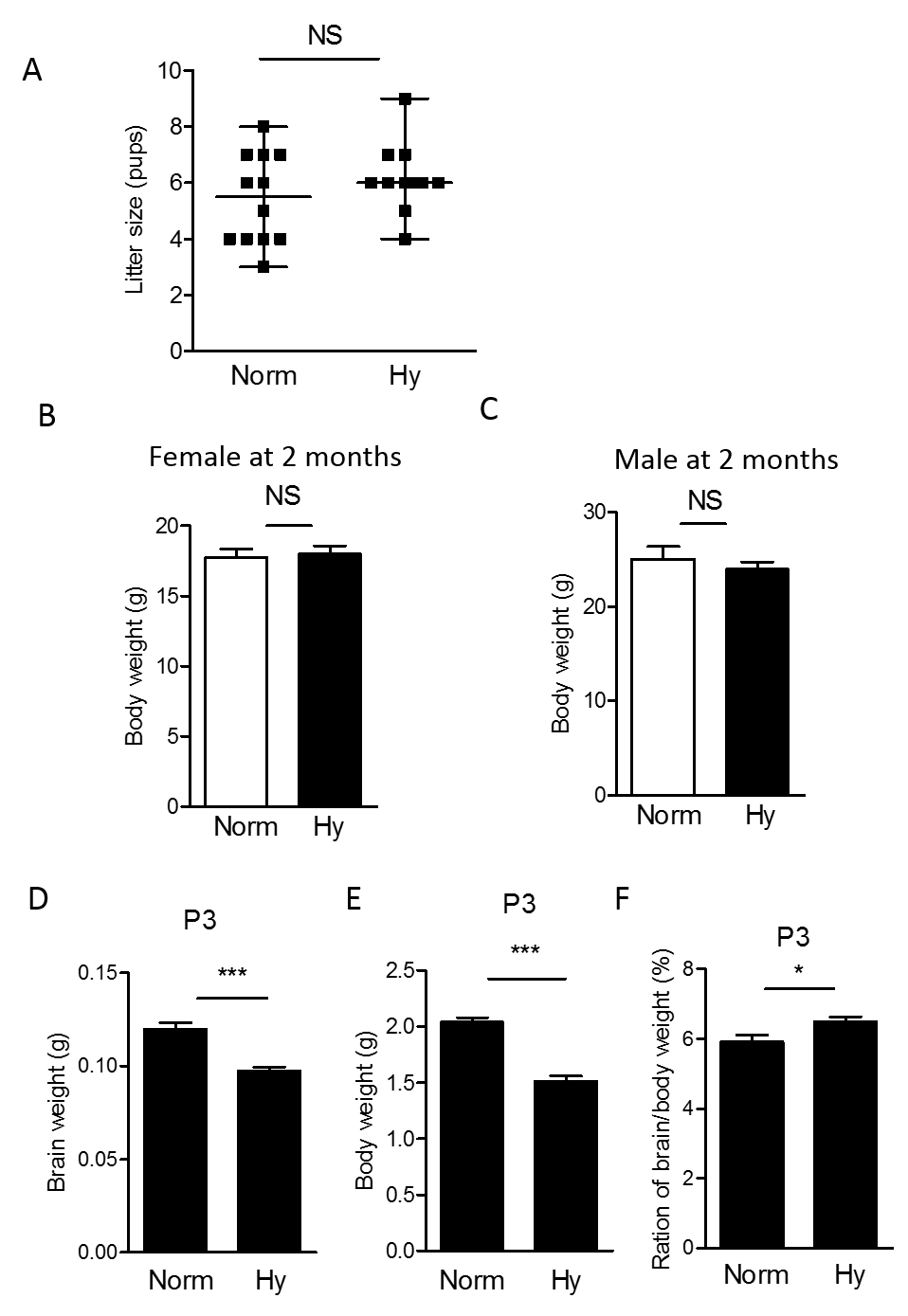


**Figure S1: Effect of gestational hypoxia on growth restriction in 5xFAD offspring.**


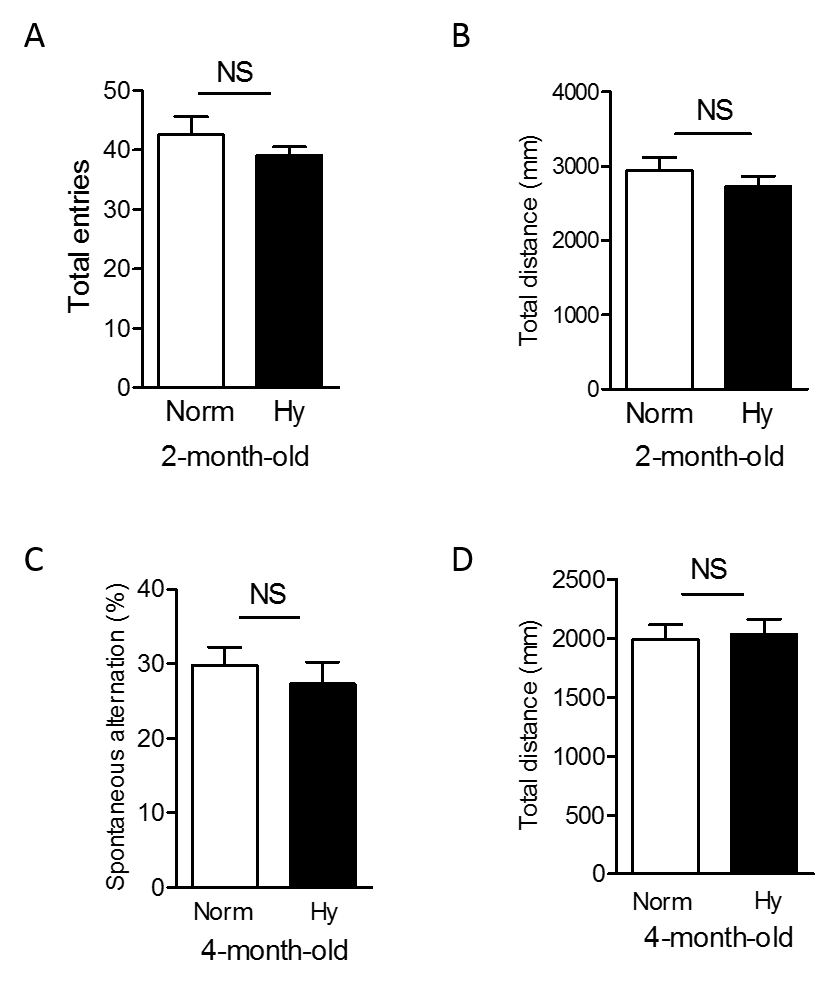


**Figure S2: Effect of gestational hypoxia on motor functions in 5xFAD offspring by Spontaneous Y-maze (sp-Y) test.**


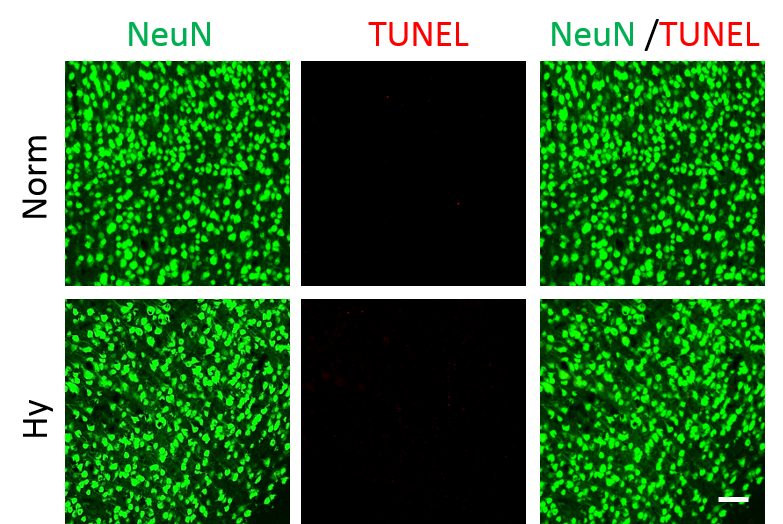


**Figure S3:The effect of gestational hypoxia on neuronal loss in the brain cortex of 5xFAD offspring.**
